# A tropical stratopause precursor for sudden stratospheric warmings

**DOI:** 10.1038/s41598-022-06864-7

**Published:** 2022-02-21

**Authors:** N. Koushik, K. Kishore Kumar, M. Pramitha

**Affiliations:** 1 Space Physics Laboratory, Vikram Sarabhai Space Centre, Indian Space Research Organisation, Thiruvananthapuram, 695022 India; 2 Present Address: Department of Physics and Astronomy, Clemson University, Clemson, SC 29634 USA

**Keywords:** Atmospheric dynamics, Climate sciences

## Abstract

Dramatic meteorological phenomena in the winter polar stratosphere known as Sudden Stratospheric Warming (SSW) events are well recognized for their impacts felt across the whole atmosphere. Apart from the influence of tropospheric forcing and stratospheric control, many studies have addressed the possible role of external factors on the occurrence of SSW events. Here, with the help of reanalysis datasets, we present a hitherto unexplored connection between the tropical upper stratosphere and the polar vortex. We identify enhanced planetary wave driving around the tropical stratopause and poleward progression of the zero-wind line as early indicators for the occurrence of SSW events. We demonstrate that the poleward progression of the zero wind line results in efficient focusing of planetary waves into the polar vortex which culminates in its disruption. Statistically, nearly 70% of the SSW events that took place so far have been preceded by enhanced tropical stratopause wave driving which points towards identifying this as a potential precursor for the occurrence of SSW events. After the year 2000, significantly a greater number of SSW events have been found to be preceded by enhanced tropical stratopause wave driving.

## Introduction

Short-term variability of the extratropical stratosphere in winter is predominantly determined by the dynamical forcing from large scale quasi-stationary planetary/Rossby waves originating from the troposphere^1,2^. Occasionally, this forcing becomes sufficiently strong enough to retard or reverse the pre-existing eastward wind flow in the stratosphere called the Polar Vortex^3,4^. Such episodes of deceleration of the mean flow are accompanied by a sudden increase in temperature, creating summer-like conditions in the winter stratosphere^5,6^. Sudden Stratospheric Warming (SSW) events as they are commonly called, have been found to significantly affect near-surface weather over polar and middle latitudes in weeks or months to follow^7–10^. Owing to this strong stratosphere-troposphere coupling, SSW events are regarded as a source of improved predictability at sub-seasonal to seasonal (S2S) scales^11,12^. Recent studies have indicated that the downward propagation of SSW events into the troposphere could be forecasted reasonably well in S2S models^13^ and that the strength of the warming is more important for the prediction of surface impacts^14^.

The dynamical forcing necessary to drive SSWs has been identified to be provided mainly by two mechanisms: (1) anomalously strong planetary wave (PW) forcing from the troposphere^15,16^ and (2) resonance excitation of planetary waves, wherein the stratospheric vortex undergoes preconditioning so as to modulate upward wave flux^17,18^. In addition, several external drivers such as tropical stratospheric Quasi Biennial Oscillation (QBO)^19,20^, El-Nino Southern Oscillation (ENSO)^20,21^, Madden–Julian Oscillation (MJO)^20,22^, the 11-year solar cycle^20,23,24^ and arctic snow^25,26^ have been identified to influence the occurrence of SSWs by modifying planetary wave forcing.

The Holton-Tan^27^ mechanism has been widely invoked to explain the control of the polar vortex by the tropical stratospheric QBO^28,29^. As per this classical mechanism, weak vortex states during the easterly phase of the QBO are attributed to the presence of the zero-wind line in the subtropics of the winter hemisphere, which facilitates the focusing of planetary waves into the polar vortex^19,24,30^. While the Holton-Tan relationship is robustly seen in many observations and model simulations, it is not clear as to what level of the QBO exerts the maximum influence on the polar vortex. More generally, it is unclear whether the location of the zero wind line plays a major role and if so at what levels^19^. Earlier studies have also demonstrated the QBO-vortex coupling using mechanisms different from the original Holton-Tan mechanism^30,31^ Many studies have suggested that the control of the polar vortex is not just limited to a particular level of the QBO, rather winds in an extended layer of the tropical stratosphere have a major role to play^32,33^. Specifically, winds around the tropical stratopause region, where the Semiannual Oscillation (SAO) is dominant, is found to significantly affect the strength of the vortex in the mid-late winter period^34–36^. In a very recent study, constraining winds in the tropical upper stratosphere in addition to the QBO region was found to significantly improve the prediction of SSW events, including its vertical structure and temporal evolution^37^.

Thus the influence of the tropical upper stratosphere on the strength of the polar vortex is increasingly being recognized. However, the physical mechanism through which the tropical upper stratospheric winds influence the polar vortex is still an open question. In the present study, we provide a plausible physical explanation for this influence, at least for a limited set of background conditions. We focus on the temporal evolution of the subtropical zero wind line before the SSW period to investigate its role in triggering the SSW. Further, we examine wave driving in the tropical stratopause to identify possible precursors for SSW events.

### Evolution of the subtropical zero-wind line

**Figure 1:**
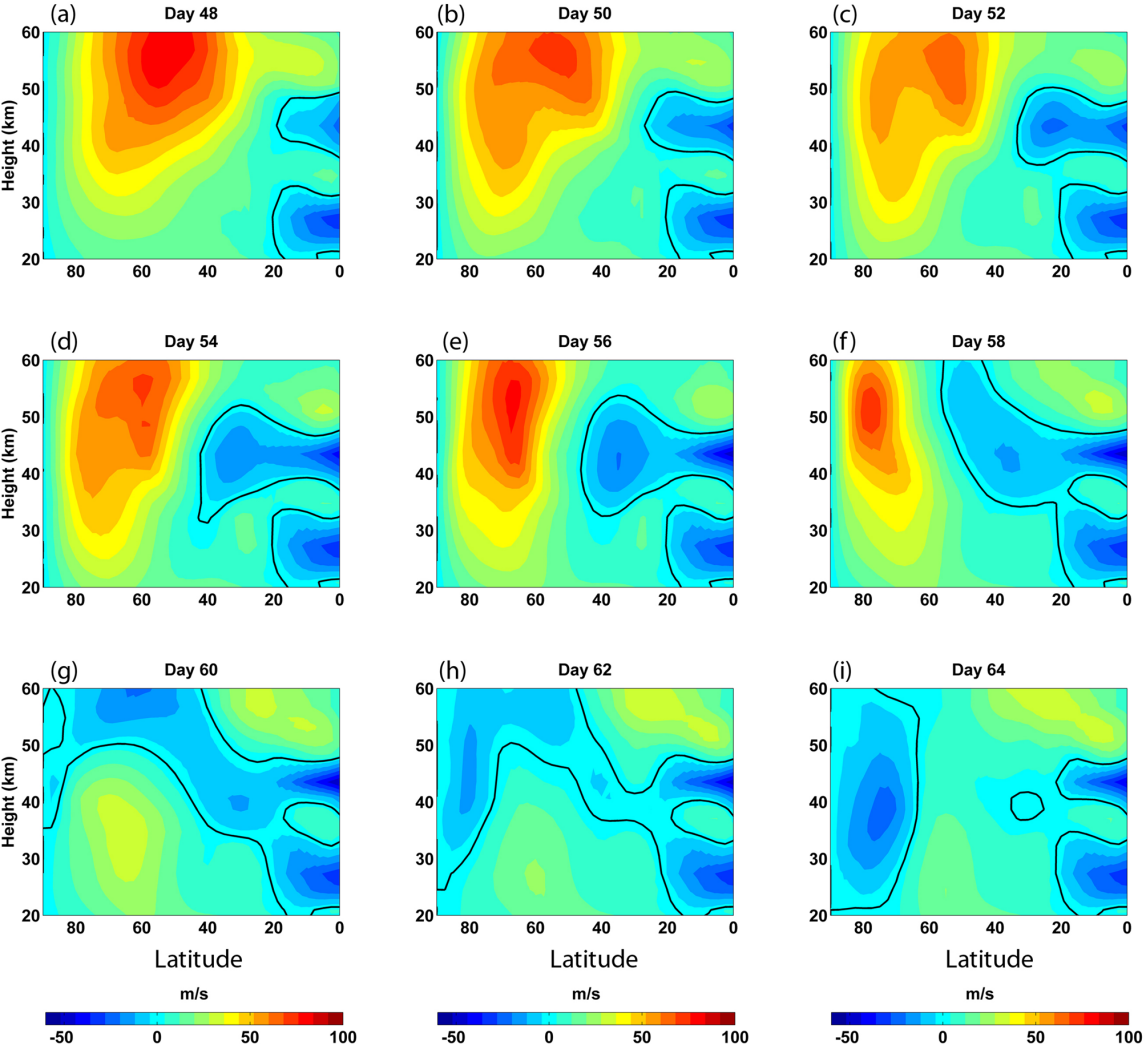
Zonal mean zonal winds (ms^−1^) in the 20–60 km altitude region over the Northern Hemisphere for (**a**) day 48, (**b**) day 50, (**c**) day 52, (**d**) day 54, (**e**) day 56, (**f**) day 58, (**g**) day 60, (**h**) day 62 and (**i**) day 64. Day 01 corresponds to 01 Dec 2000 and Day 90 Corresponds to 28 Feb 2001. Black solid lines denote the zero-wind contours. Results for the whole Dec-Feb period is given in supplementary Fig. S1. Winds in the polar cap (60° N–90° N) at 10 hPa level reverse from eastward to westward on Day 63. As per the conventional SSW definition, SSW onset for this event happens on day 73 when winds over 60° N, 10 hPa level reverse. The slow budging of the subtropical easterly wind regime around the stratopause can be noticed. Concomitantly, the region of strong westerly winds associated with the polar vortex is also found to shrink and undergo a poleward confinement. Upon reaching ~ 60° N, the zero wind line undergoes a rapid progression to the Polar Regions. Thus the poleward progression of the zero wind line starts well in advance of the SSW onset; it expands poleward and upward; upon reaching high latitudes this forms the critical line for planetary waves propagating from below. This demonstrates that the poleward propagation of the zero wind line plays a crucial part in the generation of SSW.

Using the SSW event of 2000–2001 boreal winter period as a case study, we examine the evolution of the subtropical zero wind line prior to the SSW. This is important because of the fact that the zero-wind line can act as the critical line for quasi stationary planetary waves which are largely responsible for the generation of SSW events. That is to say, these planetary waves deposit their momentum to the background winds as they encounter the zero-wind line. Zonal mean zonal winds in the 20–60 km region over the Northern Hemisphere on alternate days from day 48 to day 64 are depicted in Fig. 1 (Results for the whole Dec-Feb period are given in Supplementary Fig. S1). During the initial period, the subtropical zero wind line in the upper stratosphere is found around 20° N. As time progresses, the zero-wind line undergoes a poleward and upward excursion. The poleward excursion of the zero-wind line is driven by the deposition of easterly momentum by the interaction of planetary waves with the mean flow. Maximum poleward excursion of the zero-wind line is found on day 58, after which winds in the lower mesospheric altitudes over the Polar Regions reverse from eastward to westward. The westward wind regime further propagates downward with time, reaching stratospheric altitudes, signaling the onset of SSW. After the onset of SSW, the subtropical zero wind line retracts back to the initial position around 20° N.

### Modification of wave forcing

**Figure 2:**
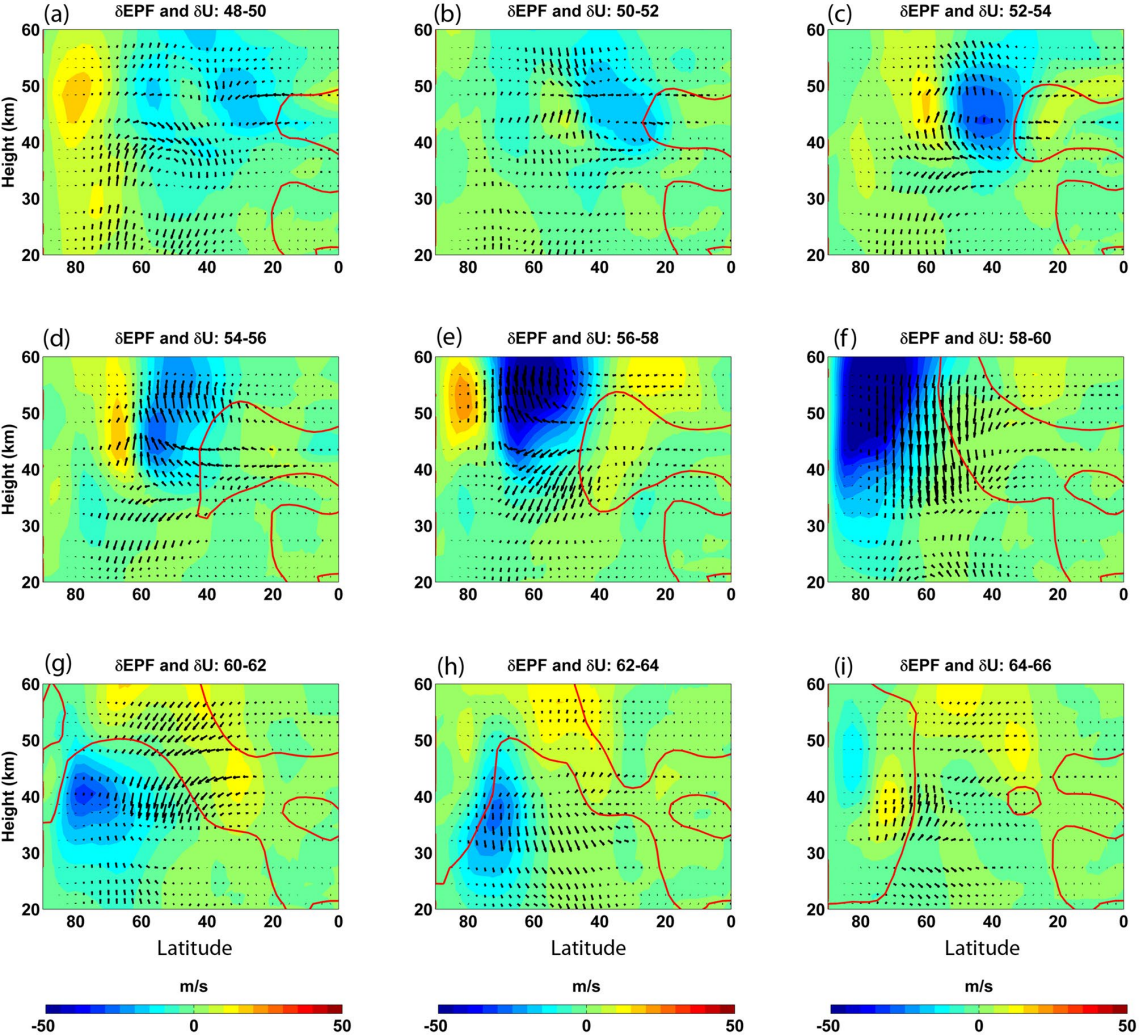
Differences in zonal mean zonal wind between alternate days shown in Fig. 1. The zero wind contours for the first day in the pair is also shown. Overlaid are the differences in EP Flux vectors between the respective days. See Supplementary Fig. S2 for the corresponding EP Flux vectors.

Eliassen-Palm (EP) diagnostics (see “Methods” section) provide an efficient way to visualize the interactions between waves and the mean-flow^38–40^, especially during extreme events such as SSWs. The effect of poleward propagating zero wind line on the wave-mean flow interaction can be inferred from Fig. 2. Starting from the initial phase, the maximum wind anomaly can be seen on the poleward flank of the subtropical zero wind line in the upper stratosphere. Moving forward in time, the wind anomaly enhances and moves poleward, together with the propagation of the zero-wind line. It can also be seen that during the phase of rapid poleward progression of the zero-wind line (Fig. 2c–e) the EP flux vectors show a prominent upward and poleward tendency. This suggests that the poleward progression of the zero-wind line facilitates the focusing of wave activity towards the polar mesosphere. Once the zero-wind line reaches the maximum poleward extent, the wind anomaly becomes the strongest, resulting from the increased wave focusing. Thereafter, the EP flux arrows point downward (Fig. 2f–h) showing the descent of the critical line and hence of the easterly wind regime. Thus, our results show that the poleward propagation of the zero-wind line helps in better focusing of planetary waves into the polar mesosphere, eventually culminating in the breakdown of the vortex through wave-mean flow interaction. (Readers are referred to Supplementary Fig. S2 for EP Flux vectors corresponding to Fig. 2).

### Tropical wave driving as a harbinger for SSW

**Figure 3:**
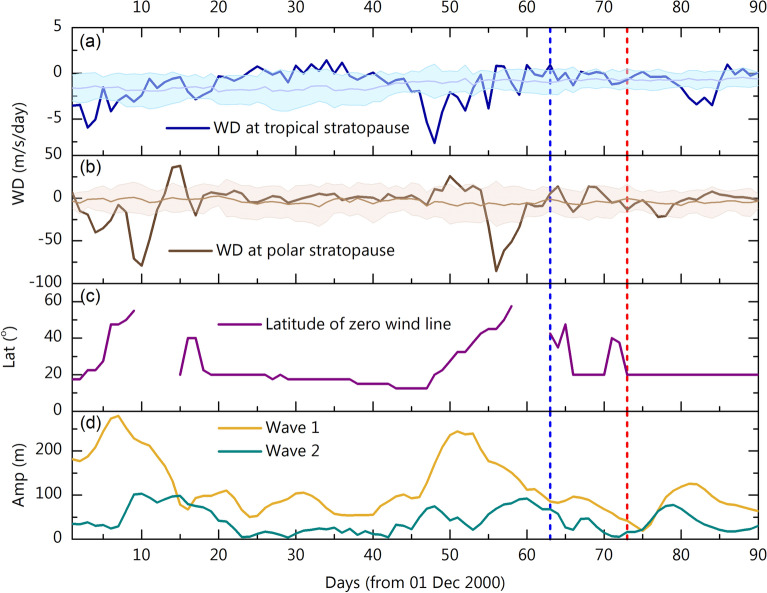
Magnitudes of wave driving in (**a**) tropical and (**b**) polar stratopause regions, (**c**) latitude of the subtropical zero wind line and (**d**) the amplitudes of wave number 1 and 2 planetary waves at 60° N, 1 hPa level during the SSW event of 2000–2001. The thin lines and the shadings in (**a**,**b**) represent the long term (1979–2021) mean wave driving and its standard deviation, respectively. Vertical blue dashed line represents the day on which winds in the polar cap at 10 hPa level reverses to westward. Red dashed line represents the day of wind reversal at 60° N, 10 hPa level. Wave driving at the stratopause is averaged for the reanalysis pressure levels 2 hPa to 0.7 hPa for tropics from 7.5 to 12.5° N (averaging to 10° N) and for polar regions from 57.5 to 62.5° N (averaging to 60° N). This averaging is done to minimize errors due to considering single latitude and pressure levels.

Having shown that the poleward propagation of the zero-wind line and subsequent focusing of the planetary waves into the polar mesosphere precedes the SSW event in the extratropical stratosphere, we now examine the temporal evolution of the wave driving (Fig. 3) to gain more physical insights. It can be seen that maximum wave driving in the polar stratopause is found around day 56. Interestingly, the maximum wave driving around the tropical stratopause peaks on day 48, approximately one week before their polar counterpart. It should be remembered that the magnitude of wave driving in the tropical stratopause is significantly smaller compared to that in the Polar Regions, as the wave driving varies inversely with the cosine of the latitude. From the long-term mean and standard deviations it can be inferred that for both the tropical as well as Polar Regions, the wave driving prior to the SSW period is significantly higher than the climatological variability. Poleward progression of the zero-wind line around day 47 coincides with the maximum tropical stratopause wave driving. Then, as the zero-wind line propagates poleward, the wave driving in the polar stratopause also increases, which also concurs with enhanced wave 1 amplitude. Finally, maximum wave driving in the polar stratopause nearly coincides with the maximum poleward extension of the zero-wind line. Winds in the polar cap reverse on day 63, which follows the peak wave driving in the polar stratopause on day 56. A minor warming event was observed around day 20 when wave 1 amplified. Prior to this minor warming also enhanced wave driving in tropical and high latitude stratopause regions can be noticed.

### Prospects/implications for prediction of SSW

From the simultaneous analysis of wave driving in the tropical and polar stratopause regions, it can be suggested that the occurrence of enhanced wave driving in the tropical stratopause prior to that over the Polar Regions can be considered as a predecessor for SSW to occur. In a similar line, we now examine multiple SSW events to confirm this observation. Results from the analysis of 29 SSW events from 1979 to 2021 are listed in Table 1. It can be seen that 20 out of the 29 SSW events considered in the study showed enhanced tropical stratopause wave driving prior to the occurrence of SSW (readers are referred to Supplementary Fig. S3 for more details). It is very interesting to note that after 2000, the number of SSW events with enhanced tropical stratopause wave driving increased significantly. Only two out of 17 SSW events from 2000 did not show enhanced stratopause wave driving prior to SSWs. Further analysis based on the phase of the QBO indicates that, while 14 of the 16 events during QBO-E showed enhanced stratopause wave driving, the same was observed in only 6 of 13 events in the QBO-W phase.

**Table 1:** Details of SSW events considered in the present study.

Sl no.	SSW event	Enhanced tropical wave driving	Phase of QBO
1	29 Feb 1980	✓	E
2	04 Mar 1981	✕	W
3	04 Dec 1981	✕	E
4	24 Feb 1984	✓	E
5	01 Jan 1985	✓	E
6	23 Jan 1987	✕	E
7	08 Dec 1987	✕	W
8	14 Mar 1988	✕	W
9	21 Feb 1989	✓	E
10	05 Feb 1995	✓	W
11	15 Dec 1998	✓	E
12	26 Feb 1999	✕	W
13	20 Mar 2000	✕	W
14	11 Feb 2001	✓	E
15	30 Dec 2001	✓	W
16	17 Feb 2002	✓	W
17	18 Jan 2003	✓	E
18	04 Jan 2004	✓	E
19	12 Mar 2005	✓	E
20	21 Jan 2006	✓	E
21	24 Feb 2007	✕	W
22	22 Feb 2008	✓	E
23	24 Jan 2009	✓	W
24	09 Feb 2010	✓	E
25	06 Jan 2013	✓	E
26	04 Mar 2016	✓	W
27	12 Feb 2018	✓	E
28	02 Jan 2019	✕	W
29	05 Jan 2021	✓	W

Central day of SSW is identified by the reversal of zonal mean zonal winds at 60° N, 10 hPa form MERRA-2 datasets. Presence of enhanced wave driving at the tropical stratopause is identified based on the climatological mean and standard deviation. Phase of QBO is identified from monthly mean equatorial zonal winds at 30 hPa.

From the foregoing analysis, it can be inferred that wave driving in the tropical stratopause region enhances prior to a significant number of SSW events. This motivates us to suggest that enhanced wave driving in the tropical stratopause can be treated as a ‘symptom’ for SSW to occur.

## Discussions

It is well established that SSW events are primarily caused by quasi-stationary planetary waves with zonal wave numbers 1 and 2 (PW1 & PW2)^5,15,41^. For very few SSW events, zonal wavenumber 3 planetary waves also may be prominent^20^. Studies have demonstrated that while wavenumber 1 events are preceded by enhanced forcing from PW1 alone, wavenumber 2 events are preceded by either PW2 alone or a combination of PW1 and PW2^42^. According to the Charney-Drazin theorem, smaller wavenumber PWs are capable of propagating to a deeper layer into the atmosphere compared to larger wavenumber PWs^43^. During the quiet period, strong winds in the mid-latitude westerly jet refract waves from below into two channels: one towards the equator and the other towards the poles^44^. Here we observe that PWs are focused more towards the equator prior to the onset of SSW. They ultimately undergo interaction with the mean flow at the low latitude stratopause region as they encounter the zero-wind line, which subsequently results in the poleward excursion of the zero-wind line. Using multiple case studies, we observe that the poleward excursion of the zero-wind line is a prominent feature. It is known that the tropical stratopause region is dominated by the semiannual oscillation (SAO) with westerly winds during equinoxes and easterly winds during solstices. The easterly SAO winds in solstices develop as a response to the dissipation of planetary waves in the winter hemisphere. Our results suggest that anomalous planetary wave dissipation prior to SSW events reflects as the poleward excursion of the zero-wind line near the SAO region. We also caution that this mechanism is found to hold true only for events preceded by enhanced planetary wave forcing. Poleward propagation of the zero wind line or enhanced tropical stratopause wave driving may not necessarily be characteristic of SSW events involving resonant amplification internally in the extratropical stratosphere.

Using EP flux diagnostics, we demonstrated that the poleward propagation of the zero-wind line facilitates the focusing of planetary waves into the polar mesosphere. Such a focusing of wave activity is also found to be crucial in establishing the critical layer at polar mesosphere. In many cases, it is found that the subtropical zero wind line itself expands poleward and results in the triggering of SSW. Here we show that the equatorial upper stratosphere plays a major role than previously anticipated in focusing the wave activity towards the polar latitudes. This, according to us, follows from the linear theory wherein wavenumber 1 PWs propagate to a deeper layer, encountering the critical layer only at the subtropical upper stratosphere and culminating in its subsequent poleward expansion. Similar poleward intruding zero wind critical line have been previously noticed in observations^45^, in numerical simulations^46^, and it was suggested that such a critical line would reflect waves back to higher latitudes^47^. Here we show that the poleward propagation of the zero-wind line originates in the upper stratosphere and the exact height of this excursion can vary from event to event depending on the background conditions.

Earlier studies have emphatically shown that the accurate prediction of SSW can aid in improving the seasonal forecasting of tropospheric weather over mid and high latitudes^13,14,48,49^, and the present study identifies enhanced tropical stratopause wave driving as one of the important precursors, which has implications in predicting SSWs. Though not in all cases, this precursor is found be present in a statistically significant number of SSW events. While there is a preponderance for this precursor to occur during QBO-E, which is in line with the well-established Holton-Tan mechanism, it is not always absent during QBO-W phases. It needs to be examined under what conditions enhanced wave driving occurs at tropical stratopause and what role do the underlying QBO winds play in controlling them. Also, our analysis identified a few cases (not shown) where there was enhanced tropical stratopause wave driving and no following SSW, which indicates that the presence of this feature alone does not imply the occurrence of an SSW. Another very important scientific question worth addressing is the increased rate of occurrence of enhanced tropical stratopause wave driving for SSW events after the year 2000, which probably points towards a shift in the internal dynamics of the system.

To conclude, the present results demonstrate that the tropical stratopause region plays a major role than previously conceived in the generation of SSW events. The background wind conditions at the tropical stratopause region may be crucial for re-focusing the planetary waves towards the polar latitudes. It needs to be verified further if this also reflects in the strength of the SAO winds. Extension of this analysis to higher altitudes is expected to shed light on the formation of the critical layer in the polar upper mesosphere which is crucial for triggering SSW events. Our results support the argument of Gray et al.^37^ in that improved representation of tropical upper stratosphere can lead to improved prediction of SSW events. We suggest more studies to be oriented in this direction to further delineate the behavior of the zero-wind line for more specific tropical stratospheric wind conditions and dominant wave forcing.

## Methods

### Data

This study makes use of temperature, zonal and meridional winds from Modern Era Retrospective Reanalysis for Research and Applications -2 (MERRA-2) assimilated meteorological fields^50^. Reanalysis datasets are found to have a reasonable agreement with satellite-based winds around the stratopause region^51^. Data from 1000 to 0.1 hPa archived at 0.5° × 0.625° (latitude × longitude) have been used to examine the day-to-day evolution of zonal mean zonal winds and to perform Eliassen-Palm Diagnostics. A pressure scale height of 7 km was used to convert the data from pressure levels to the corresponding height levels. There is no universal definition for characterizing SSW events^52^. For our discussions, we use the day in which winds in the 60° N, 10 hPa level reverse from eastward to westward as the day of SSW.

### Eliassen-Palm diagnostics

We use Eliassen Palm Flux (EP Flux) diagnostics to examine planetary wave propagation and breaking during the SSW period. In the spherical geometry, the meridional and vertical components of EP Flux are defined as^53^:

$$F_{\left( \phi \right)} = - \rho_{0}\,a\, cos\phi \left( {\overline{u^{\prime}v^{\prime}} } \right)$$

$$F_{\left( z \right)} = f\rho_{0}\, a\, cos\phi \left( {\frac{{\overline{{v^{\prime}\theta^{\prime}}} }}{{\overline{{\theta_{z} }} }}} \right)$$

(See ref^39^ and ref^1^ for detailed descriptions on EP Flux. The direction of planetary wave propagation can be inferred from the orientation of EP Flux vectors. Since the meridional and vertical components of the EP Flux differs significantly in magnitude, scaling is applied before the components are plotted in the height-latitude plane. They are scaled as follows:

$$\tilde{F}_{\left( \phi \right)} = \frac{{F_{\left( \phi \right)} *cos\phi }}{a* \pi }$$

$$\tilde{F}_{\left( z \right)} = \frac{{F_{\left( z \right)} *cos\phi }}{{10^{5} }}$$

Finally, to make the EP Flux vectors visible throughout the height domain, an additional scaling factor of $\sqrt {1000/p\left( z \right)}$ is used.

The divergence of EP Flux is used to infer the interaction of planetary waves with the mean flow. It is estimated as:

$$\nabla \cdot F = \frac{1}{a\, cos\phi }\left( {F_{\left( \phi \right)} cos\phi } \right)_{\phi } + \left( {F_{\left( z \right)} } \right)_{z}$$

Remember that there is no scaling involved in the calculation of EP Flux divergence. The total wave driving is calculated from the divergence of EP Flux as

$$D = \frac{1}{{\rho_{0} a\, cos\phi }} \nabla \cdot F$$

Here u, v and $\theta$ represent zonal wind, meridional wind and potential temperature. Potential temperature is estimated from temperature $T$ and pressure $p$ as $\theta = T \left( {\frac{{p_{0} }}{p}} \right)^{0.286}$ where $p_{0}$ is the reference pressure (1000 hPa).

$\rho_{0}$ is the background density, a is the mean radius of earth and $f$ is the coriolis parameter defined as $f = 2{\Omega }sin\phi$, where ${\Omega } = 7.29 \times 10^{ - 5} \;{\text{rad}}\;{\text{s}}^{ - 1}$ is the angular velocity of Earth’s rotation. Primed quantities denote deviations from the zonal mean and subscripts denote derivatives with respect to the subscripted variable. Over-bars represent zonal means.

Phase of equatorial QBO has been identified from the zonal mean zonal winds at 30 hPa level obtained from NOAA-PSL (https://psl.noaa.gov/data/climateindices/list/) as computed from NCEP-NCAR reanalysis.

## Supplementary Information

Supplementary Information 1.

Supplementary Information 2.

## References

[1] Andrews DG, Holton JR, Leovy CB (1987). Middle Atmospheric Dynamics.

[2] Limpasuvan V, Thompson DWJ, Hartmann DL (2004). The life cycle of the Northern Hemisphere sudden stratospheric warmings. J. Clim..

[3] Schoeberl MR (1978). Stratospheric warmings: Observations and theory. Rev. Geophys. Space Phys..

[4] Holton JR (1980). The dynamics of sudden stratospheric warmings. Annu. Rev. Earth Planet Sci..

[5] Baldwin MP, Ayarzagüena B, Birner T, Butchart N, Butler AH, Charlton-Perez AJ (2021). Sudden stratospheric warmings. Rev. Geophys..

[6] Pedatella NM, Chau JL, Schmidt H, Goncharenko LP, Stolle C, Hocke K, Harvey VL, Funke B, Siddiqui T (2018). How sudden stratospheric warming affects the whole atmosphere. Eos Earth Space Sci. News.

[7] Baldwin MP, Dunkerton TJ (2001). Stratospheric harbingers of anomalous weather regimes. Science.

[8] Thompson DWJ, Baldwin MP, Wallace JM (2002). Stratospheric connection to Northern Hemisphere wintertime weather: Implications for prediction. J. Clim..

[9] Kidston J, Scaife AA, Hardiman SC, Mitchell DM, Butchart N, Baldwin MP, Gray LJ (2015). Stratospheric influence on tropospheric jet streams, storm tracks and surface weather. Nat. Geosci..

[10] Domeisen DIV, Butler AH (2020). Stratospheric drivers of extreme events at the Earth’s surface. Commun. Earth Environ..

[11] Sigmond M, Scinocca JF, Kharin VV, Shepherd TG (2013). Enhanced seasonal forecast skill following stratospheric sudden warmings. Nat. Geosci..

[12] Tripathi OP, Baldwin M, Charlton-Perez A, Charron M, Eckermann SD, Gerber E, Harrison RG, Jackson DR, Kim BM, Kuroda Y, Lang A, Mahmood S, Mizuta R, Roff G, Sigmond M, Son SW (2015). The predictability of the extratropical stratosphere on monthly time-scales and its impact on the skill of tropospheric forecasts. Q. J. R. Meteorol. Soc..

[13] Karpechko AY, Charlton-Perez A, Balmaseda M, Tyrrell N, Vitart F (2018). Predicting sudden stratospheric warming 2018 and its climate impacts with a multimodel ensemble. Geophys. Res. Lett..

[14] Rao J, Garfinkel CI, White IP (2020). Predicting the downward and surface influence of the February 2018 and January 2019 sudden stratospheric warming events in subseasonal to seasonal (S2S) models. J. Geophys. Res.: Atmosp..

[15] Matsuno T (1971). A dynamical model of the stratospheric sudden warming. J. Atmos. Sci..

[16] Sjoberg JP, Birner T (2012). Transient tropospheric forcing of sudden stratospheric warmings. J. Atmos. Sci..

[17] Plumb RA (1981). Instability of the distorted polar night vortex: A theory of stratospheric warmings. J. Atmos. Sci..

[18] de la Cámara A, Albers JR, Birner T, Garcia RR, Hitchcock P, Kinnison DE, Smith AK (2017). Sensitivity of sudden stratospheric warmings to previous stratospheric conditions. J. Atmos. Sci..

[19] Anstey JA, Shepherd TG (2014). High-latitude influence of the quasi-biennial oscillation. Q. J. R. Meteorol. Soc..

[20] Rao J, Garfinkel CI, Chen H, White IP (2019). The 2019 New Year stratospheric sudden warming and its real-time predictions in multiple S2S models. J. Geophys. Res.: Atmosp..

[21] Domeisen DIV, Garfinkel CI, Butler AH (2019). The teleconnection of El Niño southern oscillation to the stratosphere. Rev. Geophys..

[22] Garfinkel CI, Feldstein SB, Waugh DW, Yoo C, Lee S (2012). Observed connection between stratospheric sudden warmings and the Madden-Julian Oscillation. Geophys. Res. Lett..

[23] Labitzke K, Loon HV (1988). Associations between the 11-year solar cycle, the QBO and the atmosphere. Part I: The troposphere and stratosphere in the northern hemisphere in winter. J. Atmos. Terr. Phys..

[24] Gray LJ, Crooks S, Pascoe C, Sparrow S, Palmer M (2004). Solar and QBO influences on the timing of stratospheric sudden warmings. J. Atmos. Sci..

[25] Kim BM, Son SW, Min SK, Jeong JH, Kim SJ, Zhang X, Shim T, Yoon JH (2014). Weakening of the stratospheric polar vortex by Arctic sea-ice loss. Nat. Commun..

[26] Garfinkel CI, Schwartz C, White IP, Rao J (2020). Predictability of the early winter Arctic oscillation from autumn Eurasian snow-cover in subseasonal forecast models. Clim. Dyn..

[27] Holton JR, Tan H-C (1980). The influence of the equatorial quasi-biennial oscillation on the global circulation at 50 mb. J. Atmos. Sci..

[28] Gray LJ, Anstey JA, Kawatani Y, Lu H, Osprey S, Schenzinger V (2018). Surface impacts of the Quasi Biennial Oscillation. Atmos. Chem. Phys..

[29] Rao J, Garfinkel CI, White IP (2020). How does the Quasi-Biennial Oscillation affect the boreal winter tropospheric circulation in CMIP5/6 models. J. Clim..

[30] Garfinkel CI, Shaw TA, Hartmann DL, Waugh DW (2012). Does the Holton-Tan mechanism explain how the quasi-biennial oscillation modulates the Arctic polar vortex?. J. Atmos. Sci..

[31] Rao J, Garfinkel CI, White IP (2020). Impact of the quasi-biennial oscillation on the northern winter stratospheric polar vortex in CMIP5/6 models. J. Clim..

[32] Gray LJ, Drysdale EF, Dunkerton TJ, Lawrence BN (2001). Model studies of the interannual variability of the northern hemisphere stratospherics winter circulation: The role of the quasi-biennial oscillation. Q. J. R. Meteorol. Soc..

[33] Gray LJ, Sparrow S, Juckes M, O’Neill A, Andrews DG (2003). Flow regimes in the winter stratosphere of the northern hemisphere. Q. J. R. Meteorol. Soc..

[34] Gray LJ, Phipps SJ, Dunkerton TJ, Baldwin MP, Drysdale EF, Allen MR (2001). A data study of the influence of the equatorial upper stratosphere on northern-hemisphere stratospheric sudden warmings. Q. J. R. Meteorol. Soc..

[35] Gray LJ (2003). The influence of the equatorial upper stratosphere on stratospheric sudden warmings. Geophys. Res. Lett..

[36] Pascoe CL, Gray LJ, Scaife AA (2006). A GCM study of the influence of equatorial winds on the timing of sudden stratospheric warmings. Geophys. Res. Lett..

[37] Gray LJ, Brown MJ, Knight J, Andrews M, Lu H, O’Reilly C, Anstey J (2020). Forecasting extreme stratospheric polar vortex events. Nat. Commun..

[38] Eliassen A, Palm E (1960). On the transfer of energy in stationary mountain waves. Geof. Publikasjoner.

[39] Andrews DG, McIntyre ME (1976). Planetary waves in horizontal and vertical shear: The generalized Eliassen-Palm relation and the mean zonal acceleration. J. Atmos. Sci..

[40] Edmon HJ, Hoskins BJ, McIntyre ME (1980). Eliassen-Palm cross sections for the troposphere. J. Atmos. Sci..

[41] Charlton AJ, Polvani LM (2007). A new look at stratospheric sudden warmings. Part I: Climatology and modeling benchmarks. J. Clim..

[42] Bancalá S, Krüger K, Giorgetta M (2012). The preconditioning of major sudden stratospheric warmings. J. Geophys. Res. Atmos..

[43] Charney JG, Drazin PG (1961). Propagation of planetary scale disturbances from lower into the upper atmosphere. J. Geophys, Res..

[44] Shiotani M (1986). Planetary wave activity in the troposphere and stratosphere during the Northern Hemisphere winter. J. Atmos. Sci..

[45] Vineeth C, Pant TK, Kumar KK, Sumod SG (2010). Tropical connection to the polar stratospheric sudden warming through quasi 16-day planetary wave. Ann. Geophys..

[46] Dunkerton T, Hsu C-PF, McIntyre ME (1981). Some Eulerian and Lagrangian diagnostics for a model stratospheric warming. J. Atmos. Sci..

[47] McIntyre ME (1982). How well do we understand the dynamics of stratospheric warmings?. J. Meteorol. Soc. Japan. Ser. II.

[48] Domeisen DIV, Butler AH, Charlton-Perez AJ, Ayarzagüena B, Baldwin MP, Dunn-Sigouin E (2020). The role of the stratosphere in subseasonal to seasonal prediction: 1. Predictability of the stratosphere. J. Geophys. Res.: Atmosp..

[49] Butler AH, Lawrence ZD, Lee SH, Lillo SP, Long CS (2020). Differences between the 2018 and 2019 stratospheric polar vortex split events. Q. J. R. Meteorol. Soc..

[50] Gelaro R, McCarty W, Suárez MJ, Todling R, Molod A, Takacs L, Randles CA, Darmenov A, Bosilovich MG, Reichle R, Wargan K, Coy L, Cullather R, Draper C, Akella S, Buchard V, Conaty A, da Silva AM, Gu W, Kim G-K, Koster R, Lucchesi R, Merkova D, Nielsen JE, Partyka G, Pawson S, Putman W, Rienecker M, Schubert SD, Sienkiewicz M, Zhao B (2017). The Modern-Era retrospective analysis for research and applications, version 2 (MERRA-2). J. Clim..

[51] Kawatani Y, Hirooka T, Hamilton K, Smith AK, Fujiwara M (2020). Representation of the equatorial stratopause semiannual oscillation in global atmosperic reanalyses. Atmos. Chem. Phys..

[52] Butler AH, Seidel DJ, Hardiman SC, Butchart N, Birner T, Match A (2015). Defining sudden stratospheric warmings. Bull. Am. Meteorol. Soc..

[53] Sivakumar V, Morel B, Bencherif H, Baray JL, Baldy S, Hauchecorne A, Rao PB (2004). Rayleigh lidar observation of a warm stratopause over a tropical site, Gadanki (13.5° N; 79.2° E). Atmos. Chem. Phys..

