# Supplementary figures and images for: A tropical stratopause precursor for sudden stratospheric warmings
Source: Sci Rep. 2022 Feb 21;12:2937. doi: 10.1038/s41598-022-06864-7 (PMC8861060; doi:10.1038/s41598-022-06864-7)

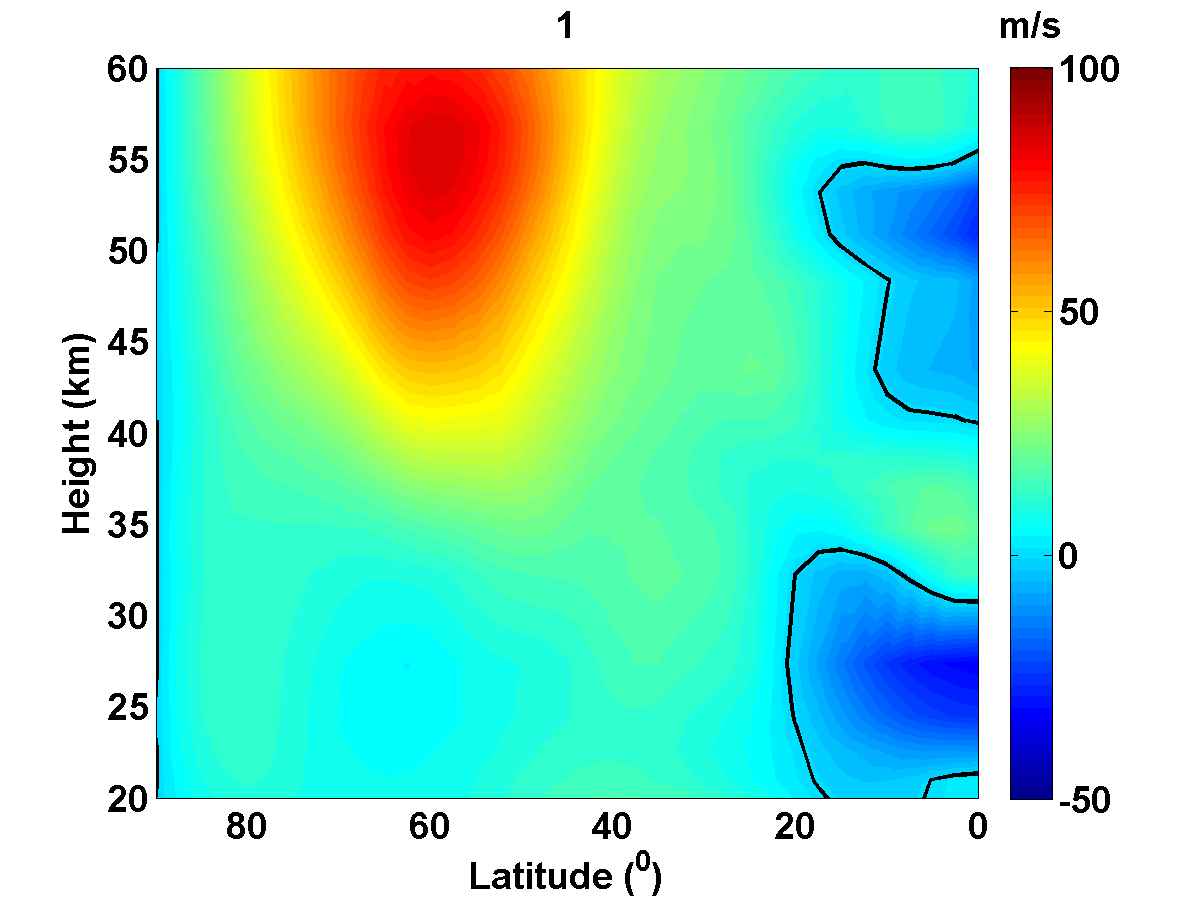

Supplement: Supplementary file 1 — Supplementary Information 1. [file 41598_2022_6864_MOESM1_ESM.gif]
