# Supplementary material for: A tropical stratopause precursor for sudden stratospheric warmings
Source: Sci Rep. 2022 Feb 21;12:2937. doi: 10.1038/s41598-022-06864-7 (PMC8861060; doi:10.1038/s41598-022-06864-7)
Supplement: Supplementary file 2 — Supplementary Information 2. [file 41598_2022_6864_MOESM2_ESM.docx]

**Supplementary information for**

**A Tropical Stratopause Precursor for Sudden Stratospheric Warmings**

**N. Koushik^1,2*^, K. Kishore Kumar^1^, M. Pramitha^1^**

**^1^Space Physics Laboratory, Vikram Sarabhai Space Centre, Indian Space Research Organisation, Thiruvananthapuram, India-695022.**

**Now at ^2^Department of Physics and Astronomy, Clemson University, South Carolina, USA-29634**


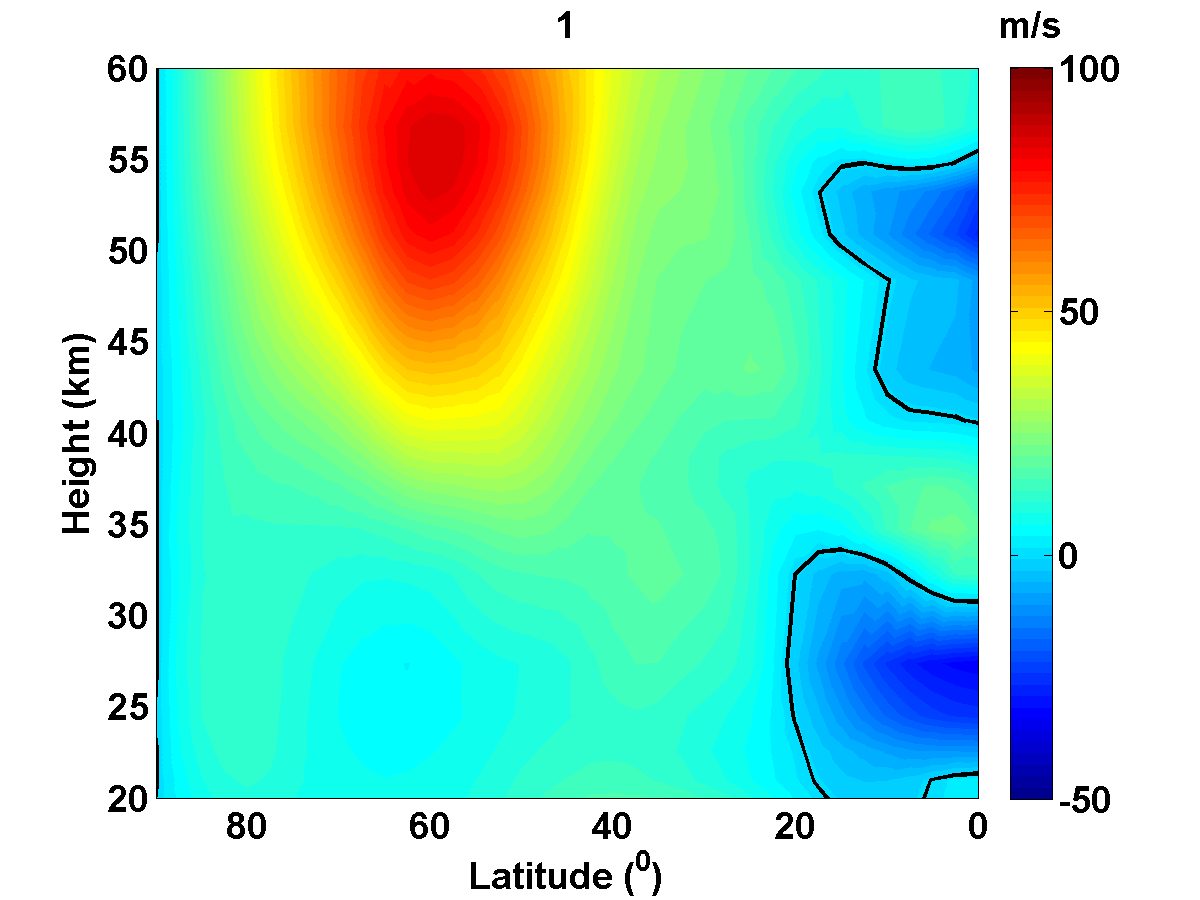


Movie S1: Evolution of zonal mean zonal winds (ms^-1^) in the 20-60 km height region for the northern hemisphere during 01 Dec 2000 to 28 Feb 2001. Black solid lines denote the zero wind contour.


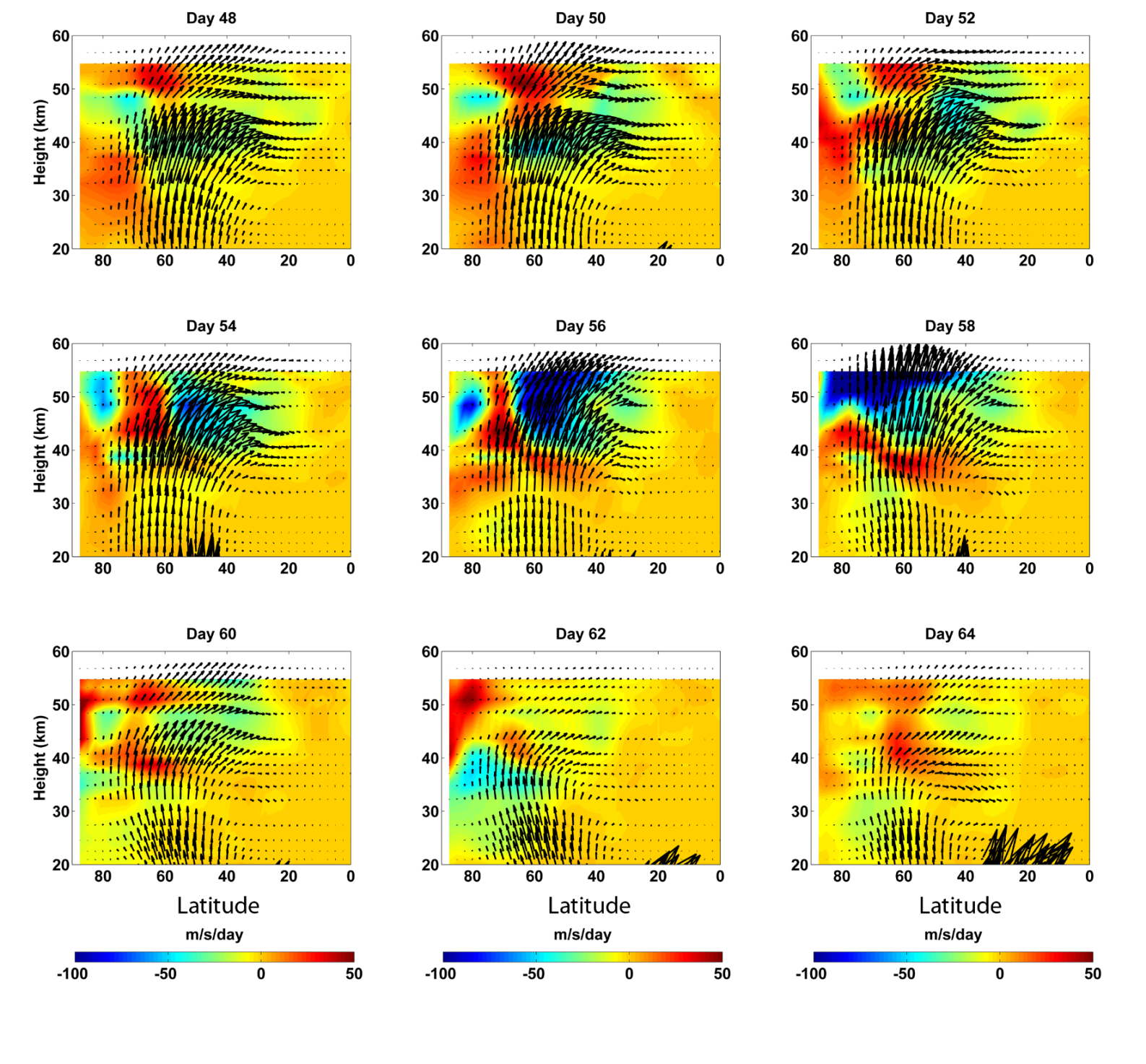


Fig S2: Evolution of wave driving (m/s/day) in the 20-60 km height region for the northern hemisphere during key stages of 2000-01 winter period. Overlaid are the EP flux vectors during the respective days.


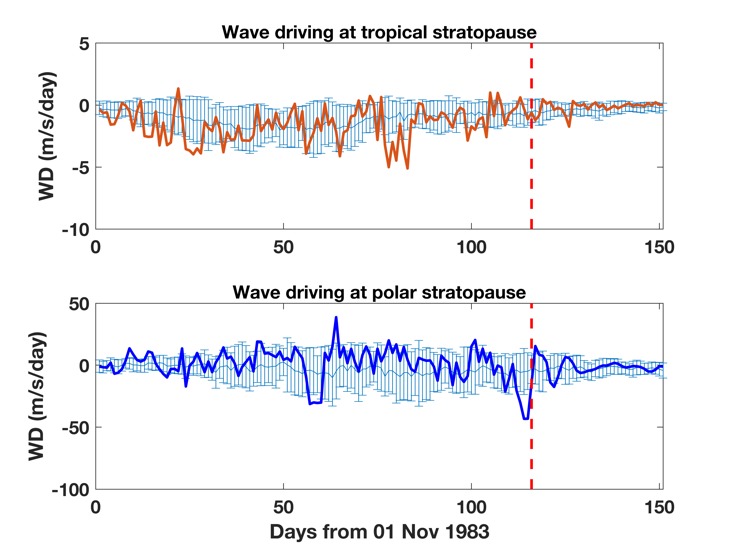

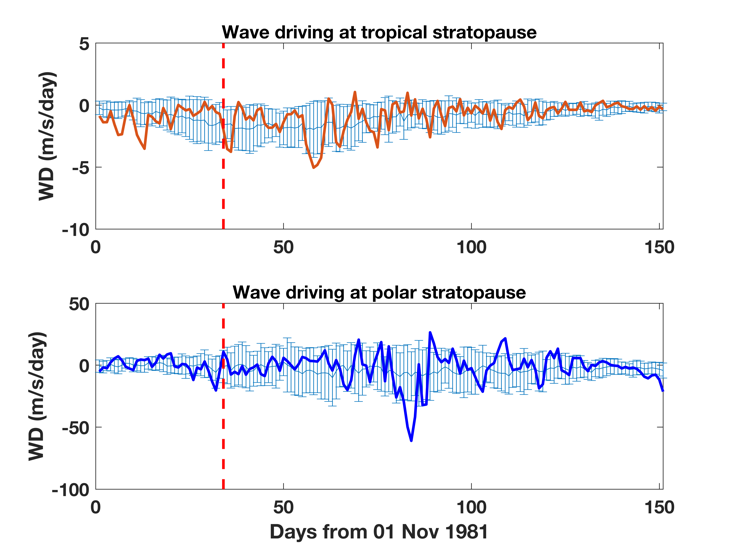

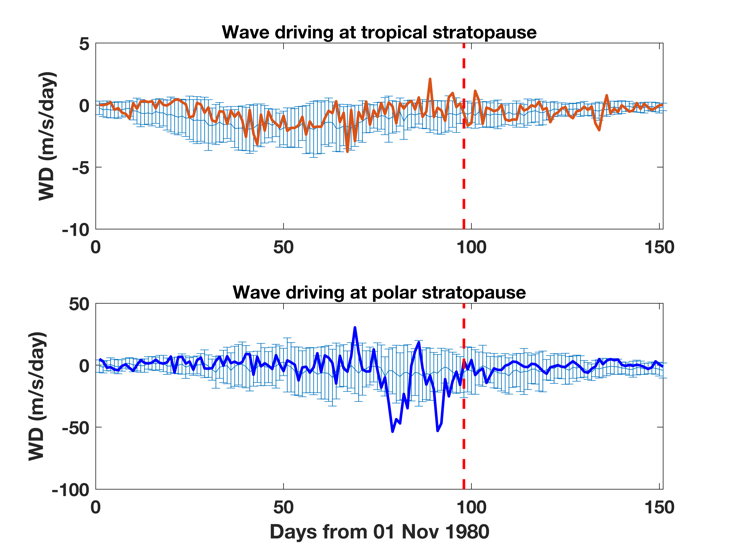

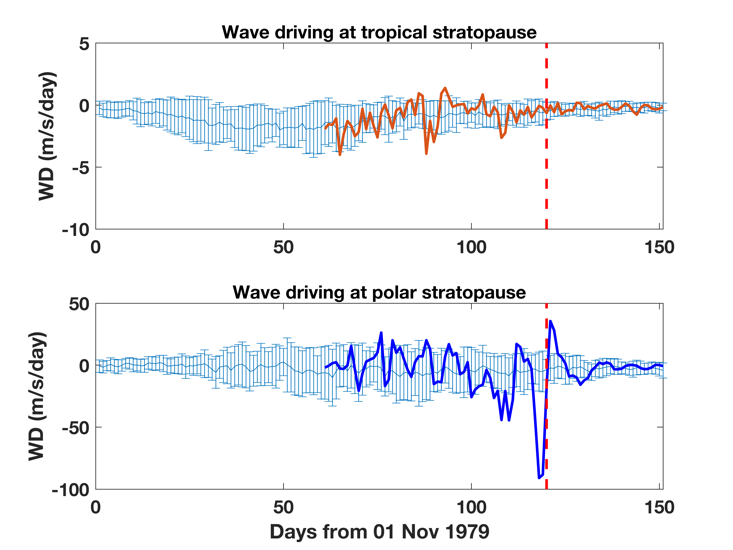


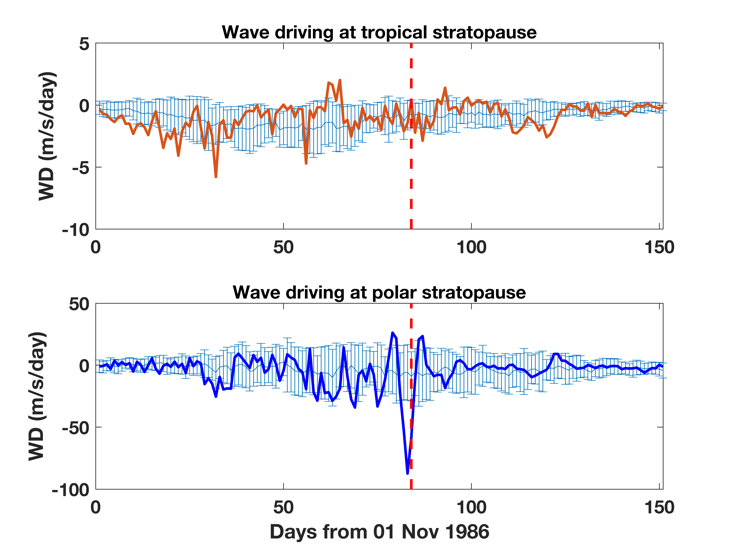

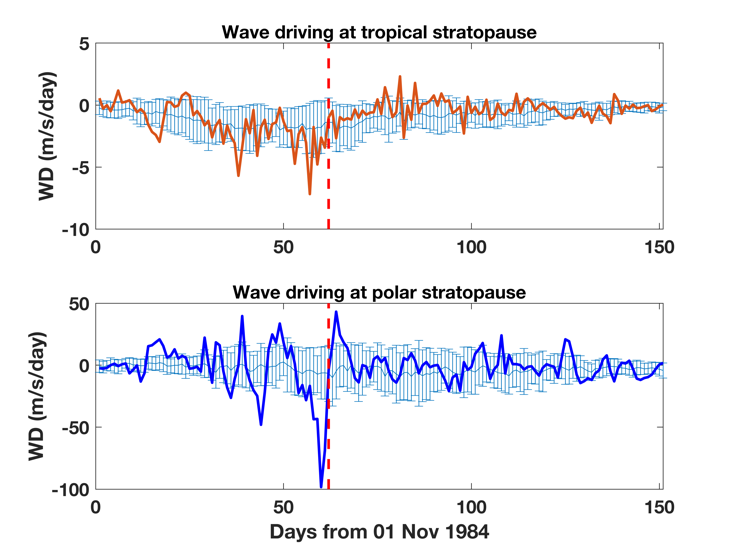


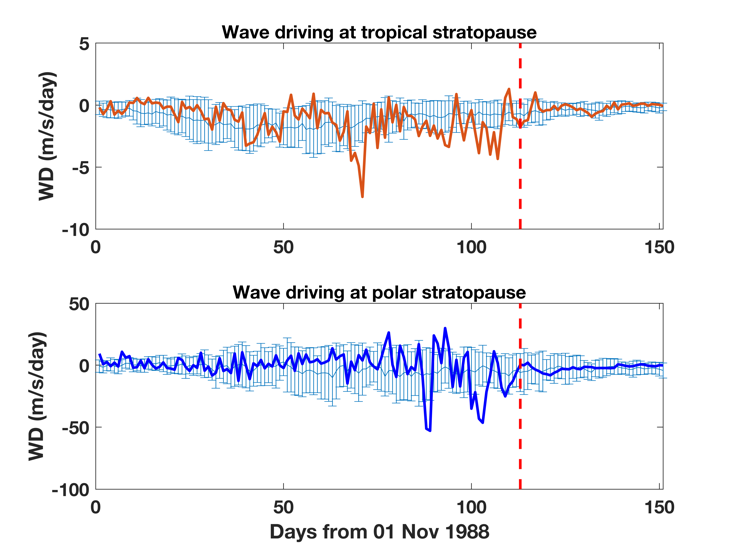

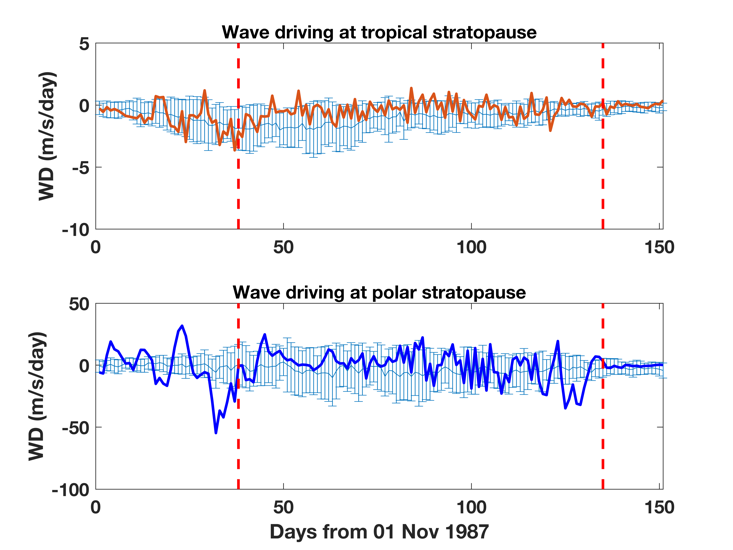


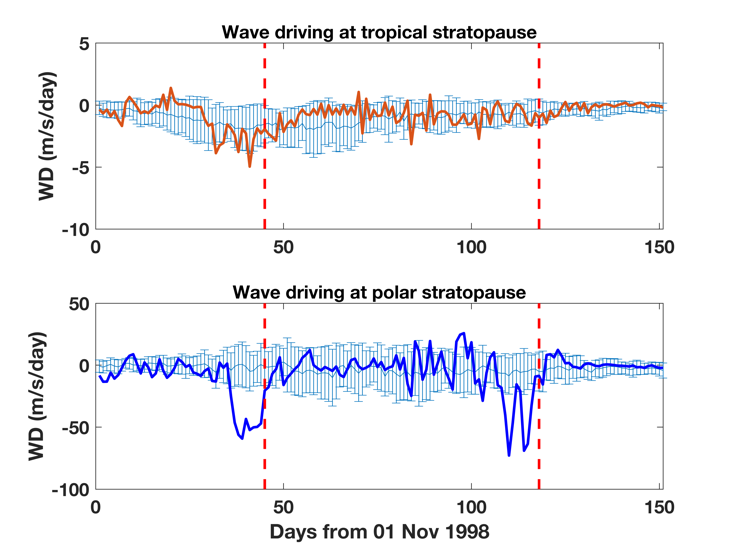

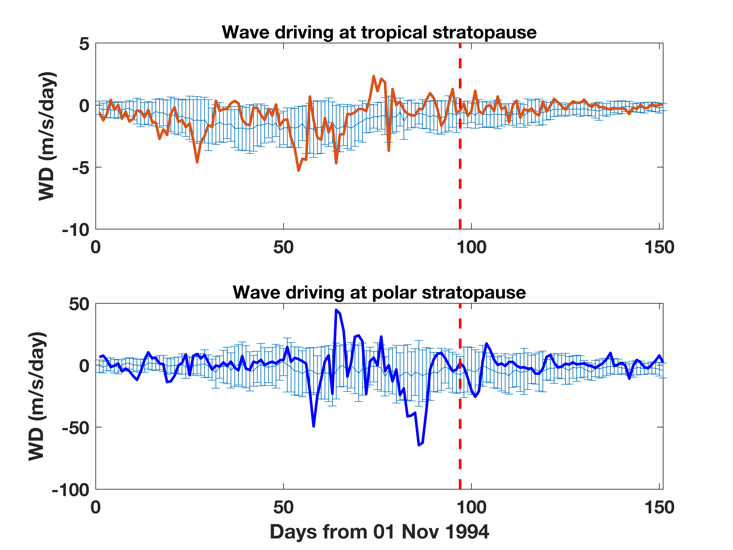

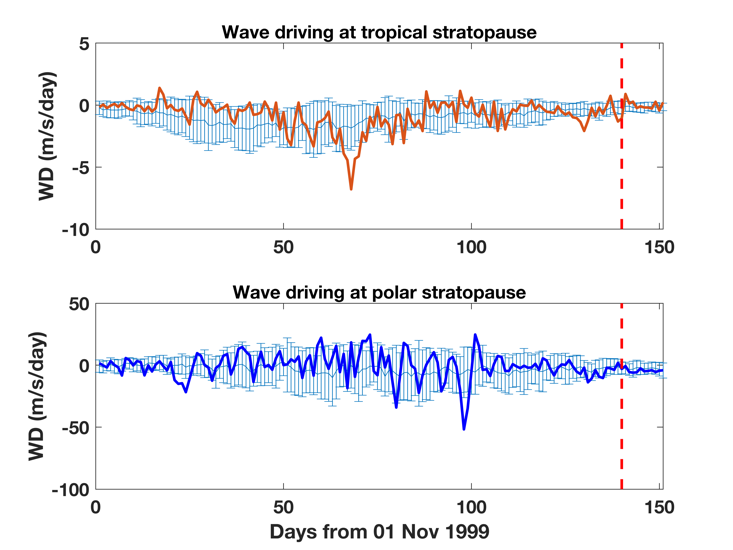


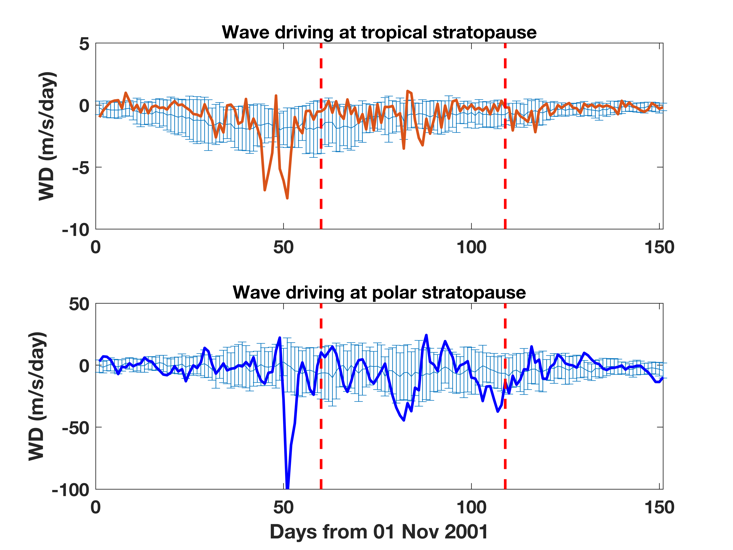


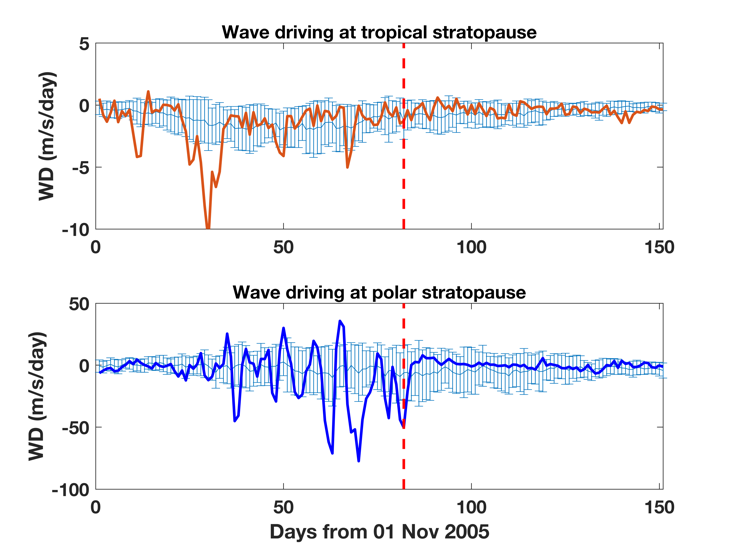

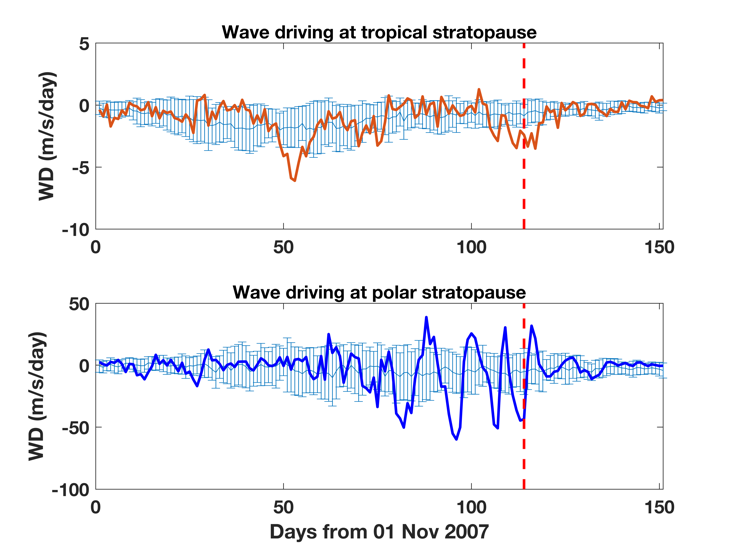

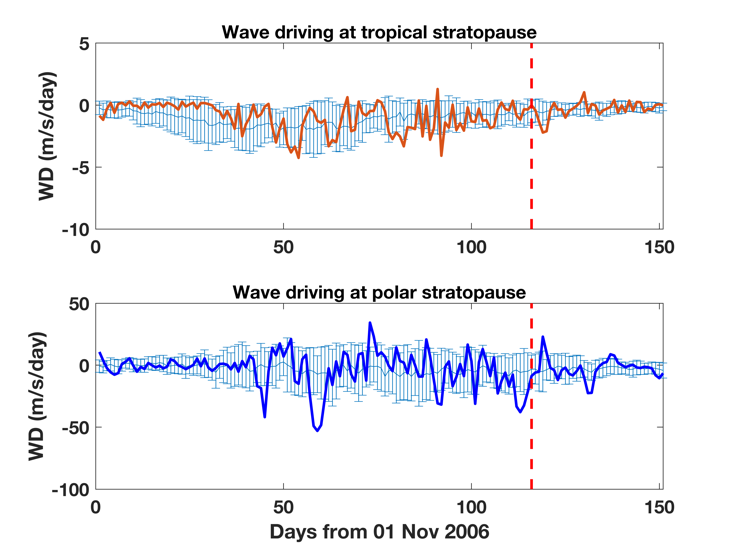

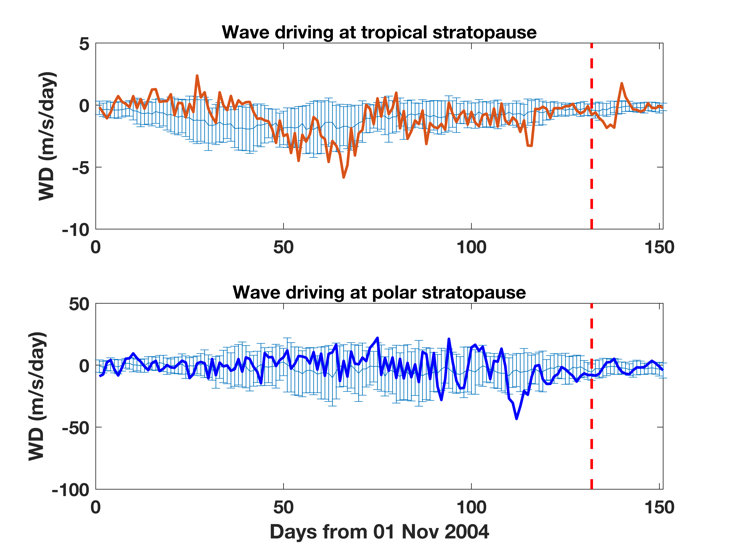

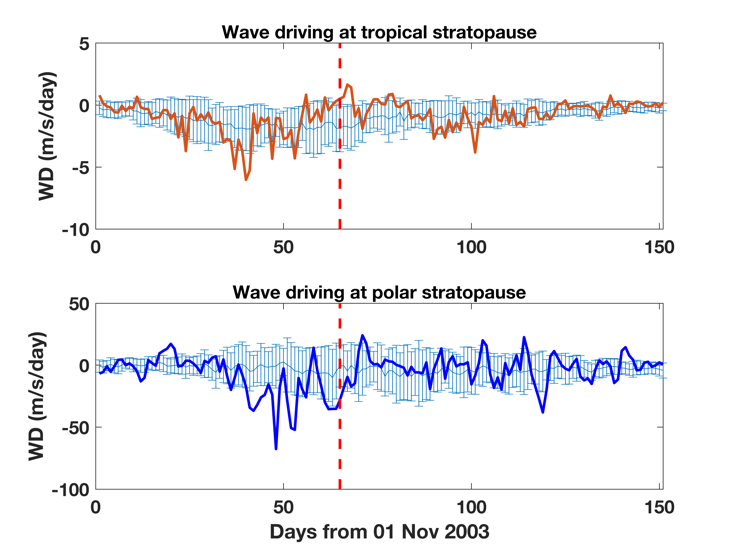

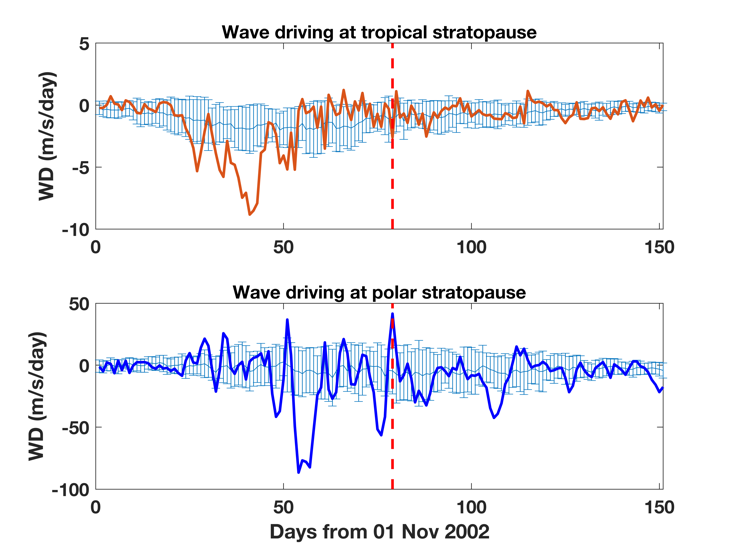


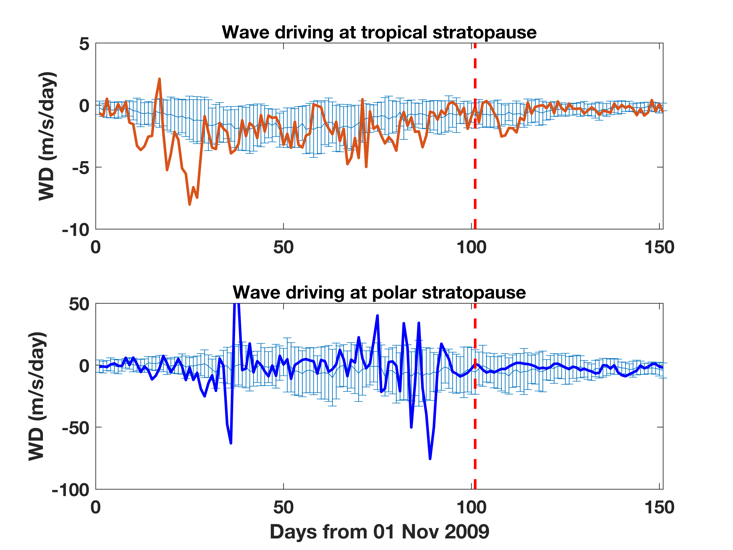

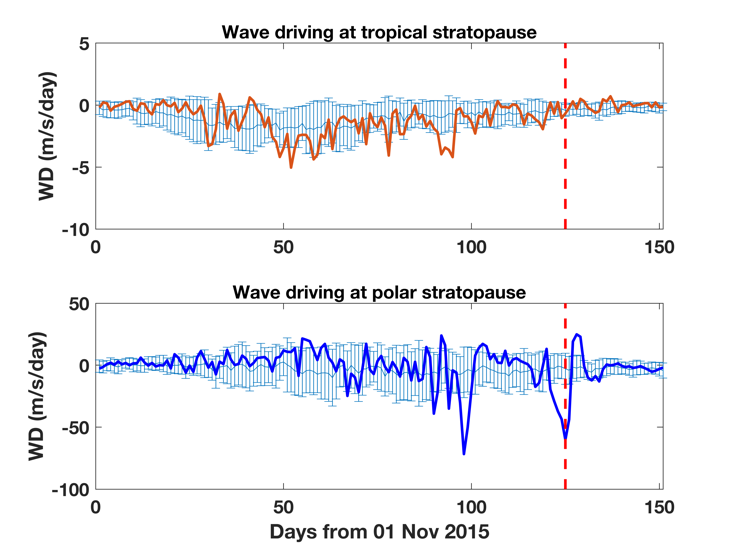

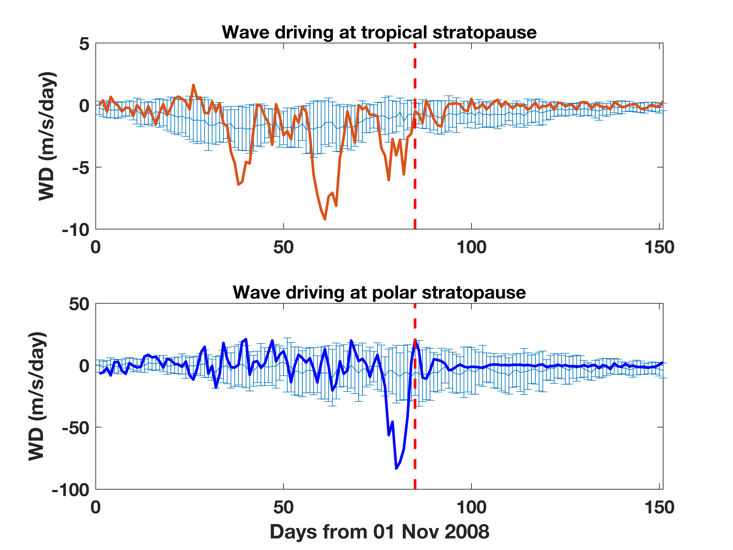


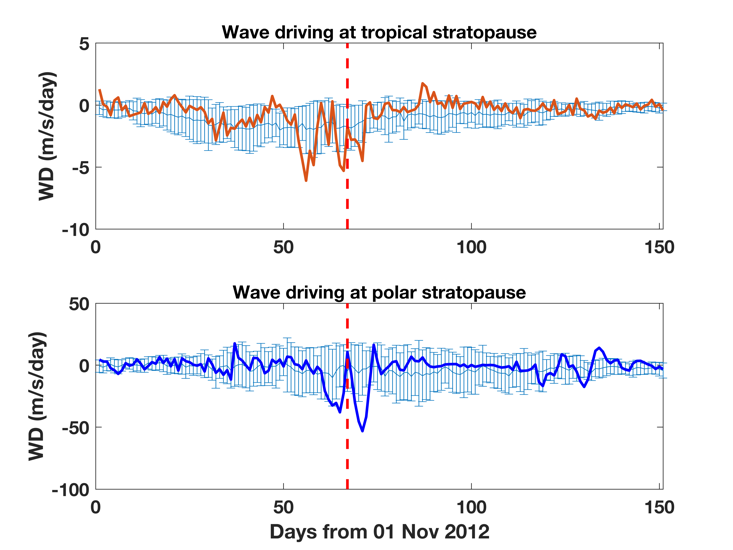


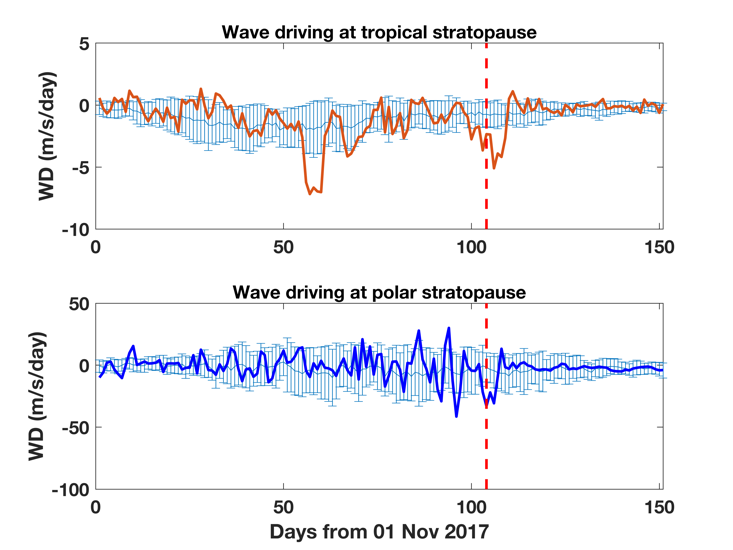

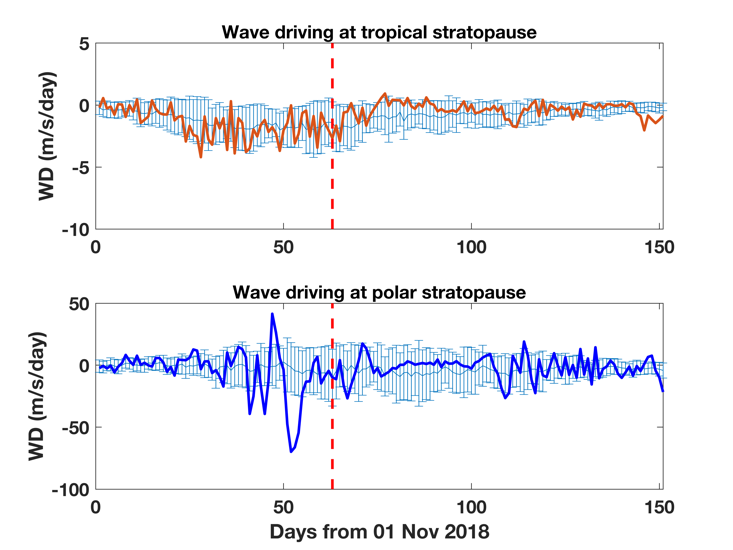


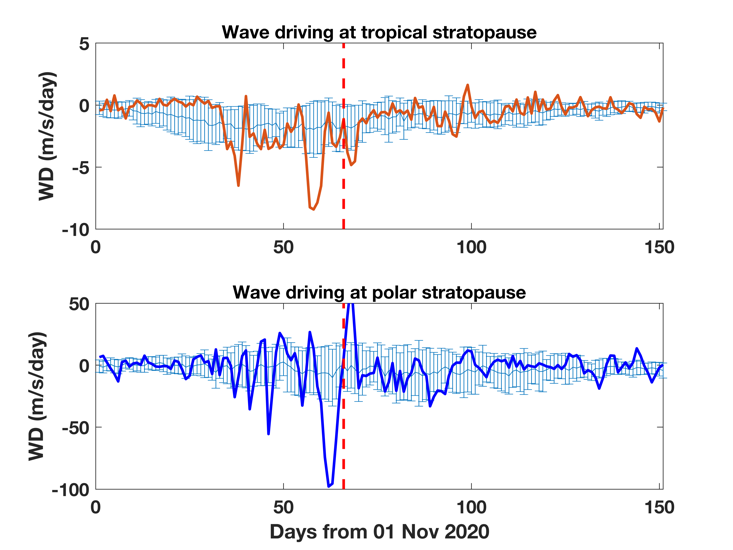


Fig S3: Wave driving (m/s/day) at the tropical (top) and polar (bottom) stratopause regions for the SSW events listed in Table 1 of the manuscript. SSW events are marked as vertical red dashed lines. The period shown is from 01 Nov to 31 Mar for all the winters.
